# Public Awareness of Cancer Symptoms, Risk Factors, and Prevention Strategies Among Adults in Poland: A Nationwide Cross-Sectional Survey

**DOI:** 10.3390/cancers18152489

**Published:** 2026-08-03

**Authors:** Kuba Sękowski, Mateusz Jankowski, Stanisław Surma, Agata Olearczyk, Wojciech S. Zgliczyński, Justyna Grudziąż-Sękowska

**Affiliations:** 1 School of Public Health, Centre of Postgraduate Medical Education, 01-826 Warsaw, Poland; 2 Department of Internal Medicine and Clinical Pharmacology, Medical University of Silesia, 40-752 Katowice, Poland; 3 National Centre for Health Policy and Health Inequalities, Cardinal Stefan Wyszynski University, 01-938 Warsaw, Poland

**Keywords:** cancer awareness, health literacy, early detection of cancer, health knowledge, attitudes, practice, cancer prevention, Poland

## Abstract

**Background/Objectives**: Cancer remains a major public health challenge, and public awareness of warning signs, risk factors, and prevention methods is essential for early detection and primary prevention. This study aimed to assess cancer knowledge among adults in Poland and identify sociodemographic factors associated with self-reported awareness. **Methods**: A nationwide cross-sectional survey was conducted in January 2026 among 1087 adults in Poland using computer-assisted web interviewing (CAWI). Non-probability quota sampling was applied based on sex, age, and place of residence. **Results**: Only 12.7% of respondents reported rather high or very high cancer knowledge. The most frequently recognized warning sign was a lump, mass, or thickening (66.9%). Tobacco use (64.9%) and genetic or familial predisposition (61.8%) were the most commonly identified risk factors, whereas 41.9% recognized overweight or obesity as a cancer risk factor. Smoking cessation and participation in cancer screening programs were each identified as preventive measures by 57.5%. However, 20.8% incorrectly believed that dietary supplements protect against cancer, and 16.2% endorsed “detox” beverages as cancer-preventive. In multivariable analysis, higher education (aOR: 1.67; 95% CI: 1.13–2.46), occupational activity (aOR: 1.56; 95% CI: 1.02–2.37), personal history of cancer (aOR: 4.27; 95% CI: 2.75–6.64), and family history of cancer (aOR: 1.85; 95% CI: 1.25–2.73) were independently associated with higher self-reported cancer knowledge. **Conclusions**: The findings indicate insufficient cancer awareness among the surveyed sample of Polish adults, particularly among men, younger adults, and individuals with lower educational attainment. Targeted educational initiatives may help improve knowledge of cancer symptoms, risk factors, and prevention strategies.

## Simple Summary

Cancer is a cause of illness and death in Poland. Still, many cancers can be prevented or treated when people recognize warning signs and understand how to reduce their risk. We conducted a survey of more than 1000 adults to assess their knowledge of cancer symptoms, risk factors, and prevention. Most people reported only average knowledge. They recognized warning signs such as a lump or unexplained weight loss and linked smoking and family history with cancer, but were less aware of infection-related risks, vaccination, and the importance of screening tests. Worryingly, some participants believed that dietary supplements or detox drinks could prevent cancer. Knowledge was higher among people with more education, those in work, and those with personal or family experience of cancer. These results show that Poland needs clear, evidence-based education campaigns about cancer, especially for men, younger adults, and people with lower levels of education.

## 1. Introduction

Cancer remains one of the leading challenges in contemporary medicine and public health, accounting for a significant percentage of premature deaths and years of life lost worldwide. According to GLOBOCAN data, approximately 20 million new cancer cases and nearly 9.7 million deaths were recorded in 2022, while projections suggest that the number of new cases may increase to over 35 million annually by 2050 [1]. In Europe, cancer remains the second leading cause of death, and in Poland, it accounts for approximately one-quarter of all deaths and is the leading cause of premature mortality before the age of 65 [2]. Breast, lung, colorectal, and prostate cancers account for the highest global cancer incidence [1,3]. Epidemiological changes observed in recent decades are primarily due to population aging, urbanization, and increasing exposure to modifiable lifestyle-related risk factors [3].

Despite significant advances in molecular diagnostics, targeted therapies, and immunotherapy, the prognosis for many cancers remains strongly dependent on the stage of disease at diagnosis [4]. Early detection of cancer enables more effective treatment and is associated with improved survival. For example, five-year survival rates for colorectal cancer exceed 90% in locally limited disease, but fall below 20% in patients with distant metastases [5]. Therefore, screening programs and secondary prevention initiatives are key elements of public health strategies. Their effectiveness, however, depends not only on the organization of the healthcare system but also on the level of public health literacy, including awareness of alarm symptoms, risk factors, and cancer prevention methods [6].

Growing evidence indicates that patients’ health literacy plays a significant role in the early detection of cancer. Health literacy refers to the ability to access, understand, appraise, and apply health information to make appropriate health-related decisions [6]. Greater knowledge of cancer symptoms has been associated with earlier help-seeking behaviors [7]. Low health literacy has been associated with diagnostic delays and seeking medical consultation at more advanced stages of disease [8].

Previous studies have shown that cancer awareness differs according to sociodemographic characteristics, particularly sex, age, educational attainment, and previous personal or family experience of cancer [9,10,11,12].

Awareness of cancer risk factors is also crucial, as a significant portion of cancer cases is associated with exposure to potentially modifiable factors. It is estimated that approximately 40% of cancer cases can be attributed to environmental and behavioral factors, such as smoking, alcohol consumption, overweight and obesity, poor diet, low physical activity, and excessive exposure to UV radiation [13]. Infectious factors also play a significant role, including infection with human papillomavirus (HPV) and hepatitis B and C viruses [14,15]. Increasing attention is also being paid to the influence of epigenetic mechanisms, chronic inflammation, and environmental–genetic interactions on the process of carcinogenesis [16,17]. Knowledge of risk factors may facilitate the adoption of preventive health behaviors and increase oncological awareness in high-risk groups.

Despite the growing importance of oncology education, previous studies conducted in Poland have evaluated selected aspects of cancer awareness, including knowledge of head and neck cancers, genitourinary cancer risk factors, and cancer prevention in specific populations [9,10,11,12]. However, comprehensive nationwide data on awareness of cancer symptoms, risk factors, and prevention strategies among the general adult population remain limited. Therefore, assessing public awareness can provide valuable information to support educational activities, preventive campaigns, and public health strategies aimed at improving cancer awareness.

This study aimed to assess public knowledge of cancers, including awareness of cancer symptoms, risk factors, and prevention methods, and to identify sociodemographic factors associated with cancer awareness among adults in Poland.

## 2. Materials and Methods

### 2.1. Study Design and Population

This cross-sectional survey was conducted in January 2026 among a nationwide sample of adults in Poland. Data were collected using the Computer-Assisted Web Interview (CAWI) technique.

Based on the scientific assumptions and methods developed by the Authors, the survey was administered by the Nationwide Research Panel Ariadna [18], one of the largest online research panels in Poland, comprising over 100,000 registered and verified users. Participants were recruited using a non-probability quota sampling method based on sex, age, and place of residence, according to demographic data published by Statistics Poland (GUS) [19]. Individuals who declined to participate were replaced with respondents from the same demographic stratum to maintain the predefined quota structure. Only complete questionnaires were included in the final analysis. The inclusion criteria were age ≥ 18 years, residence in Poland, and provision of informed consent to participate in the study. Individuals who did not complete the questionnaire were excluded from the analysis.

Participation was voluntary and anonymous. Before completing the survey, all participants were informed about the study objectives and provided electronic informed consent.

Similar methodology was applied in other nationwide studies in Poland [10,20,21].

### 2.2. Measures

The questionnaire was developed after a review of the available scientific literature and previously published population-based surveys on cancer awareness [4,5,6,7,8,9,10,11,12,13]. The questionnaire items and response options were selected to cover four domains: self-reported cancer knowledge, awareness of cancer symptoms, awareness of cancer risk factors, and awareness of cancer prevention methods. It included items that assessed participants’ self-reported knowledge of cancers, their awareness of potential warning signs, cancer risk factors, and methods of cancer prevention. Participants were also asked about their sociodemographic characteristics, such as sex, age, place of residence, educational level, occupational status, marital status, and financial situation. Additional questions inquired about personal cancer history and the family history of cancer among first-degree relatives.

Self-reported knowledge of cancer was evaluated using a five-point Likert scale ranging from “very low” to “very high”. Self-reported cancer knowledge reflected participants’ subjective assessment of their own knowledge and did not represent an objective measure of cancer awareness. Awareness of cancer symptoms, risk factors, and prevention methods was assessed using multiple-choice questions, in which respondents could select all answers they believed were correct.

The questionnaire was developed based on the available literature and previously published population-based surveys on cancer awareness. Prior to the main survey, the questionnaire was pilot-tested in a group of 10 adults aged 18–72 years. To evaluate test–retest reliability, participants completed the questionnaire on two occasions separated by a 10-day interval. Following the pilot testing, minor revisions were made to improve the clarity of selected items. Agreement for the key questionnaire items was high, with Cohen’s kappa coefficients ranging from 0.72 to 0.94.

### 2.3. Statistical Analysis

Data were analyzed using IBM SPSS Statistics version 29 (IBM Corp., Armonk, NY, USA). Categorical variables were presented as frequencies and percentages. Associations between respondents’ characteristics and awareness-related variables were assessed using the chi-square test. Logistic regression analysis was performed to identify factors associated with reporting a rather high or very high level of self-reported cancer knowledge (dependent variable). In the bivariable model, each independent variable was analyzed separately. Independent variables considered potentially relevant to self-reported cancer knowledge were selected a priori for bivariable analyses based on theoretical considerations, epidemiological relevance, and evidence from previous studies. Each selected independent variable was first analyzed separately in a bivariable logistic regression model. Variables demonstrating a statistically significant association in the bivariable analyses were subsequently entered into the multivariable logistic regression model. Categories of selected variables were merged where necessary to ensure adequate cell counts and facilitate regression analyses. Results were presented as odds ratios (ORs) with 95% confidence intervals (95%CI). Statistical significance was defined as *p* < 0.05.

## 3. Results

A total of 1087 adults participated in the study. Females accounted for 50.7% of respondents, and the largest age group was participants aged 35–44 years. Most respondents had secondary or higher education, were occupationally active, and lived in urban areas. Detailed characteristics of the study population are presented in Table 1.

### 3.1. Public Awareness of Cancers

Most respondents reported a moderate level of knowledge about cancers, while only 12.7% declared a rather high or very high level of knowledge. Enlarged lymph nodes (69.5%), a lump or thickening (66.4%), and unexplained weight loss (64.7%) were the most frequently recognized warning signs of cancer. Smoking tobacco (64.9%), genetic or familial predisposition (61.8%), overweight or obesity (41.9%), and excessive UV exposure (59.2%) were the most commonly identified cancer risk factors.

Regarding cancer prevention, smoking cessation and avoidance of secondhand smoke (57.5%), participation in screening examinations (57.5%), and seeking medical attention for concerning symptoms (56.8%) were the most frequently indicated preventive measures. However, 20.8% of respondents incorrectly believed that dietary supplements protect against cancer, and 16.2% believed that “detox” beverages may prevent cancer. Detailed results are presented in Table 2.

### 3.2. Factors Associated with Self-Reported Cancer Knowledge

In multivariable logistic regression analysis, respondents with higher education had higher odds of reporting a rather high or very high level of knowledge about cancer (aOR: 1.67; 95% CI: 1.13–2.46; *p* = 0.009). Occupationally active individuals (aOR: 1.56; 95%CI: 1.02–2.37; *p* = 0.04), respondents who had been diagnosed with cancer (aOR: 4.27; 95%CI: 2.75–6.64; *p* < 0.001), and those with a family history of cancer (aOR: 1.85; 95%CI: 1.25–2.73; *p* = 0.002) also had higher odds of reporting higher levels of cancer knowledge. Moreover, divorced or widowed respondents had higher odds of reporting a rather high or very high level of cancer knowledge compared to single individuals (aOR: 2.97; 95%CI: 1.32–6.69; *p* = 0.008). Detailed results are presented in Table 3.

### 3.3. Sociodemographic Differences in Awareness of Cancer Symptoms

There were significant sociodemographic differences in awareness of symptoms that may indicate cancer (Table 4). Women demonstrated higher awareness of most cancer warning signs compared to men. Respondents with higher education and those with a family history of cancer generally showed greater awareness of cancer symptoms than their counterparts. Awareness also varied by age group and personal history of cancer for several symptoms. In contrast, place of residence and occupational status were associated with awareness of only selected cancer symptoms. Detailed results are presented in Table 4.

### 3.4. Sociodemographic Differences in Awareness of Cancer Risk Factors

There were significant sociodemographic differences in awareness of cancer risk factors (Table 5). Women more frequently identified several established cancer risk factors, particularly lifestyle-related and infection-related factors, compared to men. Respondents with higher education demonstrated greater awareness of most cancer risk factors than those without higher education. Awareness also varied by age, self-reported financial situation, personal history of cancer, and family history of cancer. Individuals with a family history of cancer generally showed higher awareness of established cancer risk factors. In contrast, respondents without such a history more often reported uncertainty or lower recognition of selected risk factors. Detailed results are presented in Table 5.

### 3.5. Sociodemographic Differences in Awareness of Cancer Prevention Methods

There were significant sociodemographic differences in awareness of cancer prevention methods (Table 6). Women more frequently identified evidence-based cancer prevention strategies than men. Respondents with higher education generally demonstrated greater awareness of preventive measures, including lifestyle modifications, participation in screening programs, and vaccination. Awareness of selected prevention methods also varied by age, occupational status, personal history of cancer, and family history of cancer. Individuals with a family history of cancer were more likely to recognize established cancer prevention strategies. Detailed results are presented in Table 6.

## 4. Discussion

This nationwide cross-sectional survey assessed public knowledge of cancers among adults in Poland, including awareness of cancer warning signs, risk factors, and prevention methods, and identified sociodemographic factors associated with self-reported cancer knowledge.

Most respondents rated their cancer knowledge as moderate, and only 12.7% declared a rather high or very high level, indicating that self-perceived cancer knowledge remains limited in the general adult population. Similarly, in a nationwide Polish survey on head and neck cancers, 30% of respondents reported knowing nothing about these cancers and 40.8% reported knowing very little [9]. Consistently, Sokołowska et al. [10] demonstrated limited general knowledge of cancer in a regional Polish study, and Berbecka et al. [11] reported low awareness of gastrointestinal malignancies in a provincial population. Together, these findings suggest that suboptimal cancer knowledge is a persistent issue across different cancer types and regions in Poland rather than an isolated phenomenon confined to a single cancer site or locality [9,10,11].

In our study, 20.8% of respondents incorrectly endorsed dietary supplements as cancer-protective, and 16.2% believed that “detox” beverages may prevent cancer, highlighting the presence of specific misconceptions that may undermine evidence-based prevention efforts. Similar patterns have been reported in other countries, where population-based surveys demonstrate limited cancer awareness and widespread misconceptions about cancer risk factors [22]. In a nationwide Irish study [23], only 52% of adults correctly identified at least one major modifiable cancer risk factor, while many participants overestimated the protective value of vitamins and supplements and endorsed various “myths” such as detox diets or specific foods preventing cancer. These convergent findings underscore the need for targeted health education that not only improves general knowledge but also explicitly addresses misinformation regarding purportedly “protective” products and practices [9,10,11,22,23].

In multivariable logistic regression, higher education, occupational status, personal cancer diagnosis, family history of cancer, and divorced or widowed marital status were independently associated with a higher self-reported cancer knowledge level. Higher educational attainment was also identified as a key determinant of cancer awareness in prior Polish nationwide CAWI-based surveys on head and neck cancers and genitourinary cancer risk factors [10,24]. The exceptionally strong association with personal cancer diagnosis likely reflects direct exposure to cancer-related medical information during the diagnostic and treatment process. Family history of cancer—reported by 49.9% of respondents—was similarly associated with higher awareness in the genitourinary cancer awareness study in Poland [25]. The association with divorced or widowed marital status may reflect the higher mean age in this group and consequently greater lifetime exposure to cancer-related health events.

Comparable sociodemographic patterns in self-reported cancer knowledge have been demonstrated in population-based studies outside Poland. In a US survey of adults aged ≥ 50 years, lower educational attainment consistently predicted poorer recognition of cancer symptoms in multivariable analyses, confirming education as a key determinant of cancer awareness in another high-income setting [25]. Similarly, analyses of the Spanish Onco Barometer 2020 demonstrated that individuals from lower socioeconomic groups were more likely to report not knowing how lifestyle factors influence cancer risk, and that low perceived influence clustered among groups at higher cancer risk [26]. Recent European research has further documented substantial educational disparities in awareness of modifiable cancer risk factors, reinforcing the central role of education in shaping cancer-related knowledge across different countries [27]. These convergent findings suggest that the strong association between higher education and cancer knowledge observed in our study reflects a broader international pattern rather than a Poland-specific phenomenon.

There were marked sociodemographic differences in awareness of cancer warning signs. Women demonstrated consistently higher awareness of all 12 assessed cancer warning signs compared to men (all statistically significant). A similar sex difference was observed in the nationwide Polish survey on head and neck cancer awareness, in which female sex was associated with greater recognition of cancer symptoms [10]. Younger respondents (18–29 years) showed notably lower awareness of several warning signs, including unexplained weight loss, enlarged lymph nodes, and persistent cough, compared to older age groups. Respondents with higher education showed greater awareness of all assessed symptoms compared to those without higher education (all statistically significant), reinforcing the strong educational gradient observed in self-reported cancer knowledge.

These patterns are consistent with international evidence. In a US population-based survey of adults aged ≥ 50 years, lower educational attainment consistently predicted poorer recognition of multiple cancer symptoms [25]. Analyses of the Spanish Onco Barometer 2020 similarly showed that respondents from lower socioeconomic groups were more likely to report not knowing the influence of lifestyle factors on cancer risk and had lower awareness of cancer-related information overall [27]. Recent studies from Georgia [28] and Ireland [29] have likewise demonstrated that lower educational attainment and socioeconomic position are associated with poorer recognition of common cancer warning signs, indicating that social inequalities in cancer awareness are a widespread challenge across diverse health systems. Our findings therefore align with a growing body of literature suggesting that efforts to improve early cancer detection should prioritize groups with lower education and socioeconomic status, as well as younger adults and men, who consistently report lower awareness of key symptoms.

Awareness of cancer risk factors also varied substantially across sociodemographic groups. Tobacco use and genetic or familial predisposition were the most frequently identified cancer risk factors. In comparison, lifestyle-related factors—including overweight or obesity, low physical activity, and unhealthy diet—were less commonly recognized. This imbalance suggests that public understanding remains focused on a limited set of well-publicized risks, while other modifiable determinants receive comparatively less attention. The lower public recognition of lifestyle-related factors mirrors findings from a nationally representative Spanish survey, in which—except for tobacco—lifestyle factors were among the least recognized cancer risk factors, particularly among men and older individuals [27]. Alcohol as a cancer risk factor was identified by fewer than half of respondents (48.5%), which is consistent with documented low public awareness of the alcohol–cancer link in surveys conducted across 14 European countries [30] and with a scoping review of EU and UK populations [31]. Infection-related risk factors were also underrecognized, especially Helicobacter pylori (29.5%).

These findings should also be interpreted in the context of population-level exposure to modifiable cancer risk factors in Poland. According to Health at a Glance 2025, alcohol consumption in Poland remains above the OECD average, while insufficient physical activity is more common than across OECD countries overall. The same report also points to persisting challenges related to excess body weight and dietary patterns, including fruit and vegetable consumption. In this context, limited recognition of alcohol consumption, low physical activity, unhealthy diet, and excess body weight as cancer risk factors is particularly relevant. These are not marginal exposures, but common behavioral determinants of population health; therefore, gaps in awareness may weaken the perceived relevance of lifestyle-related cancer prevention and reduce public responsiveness to prevention messages [32].

The age gradient observed for selected risk factors is particularly relevant in the context of lifestyle-related prevention. Respondents aged 18–29 years were less likely than older respondents to recognize tobacco use, alcohol consumption, low physical activity, and excessive UV exposure as cancer risk factors. Although age differences were not statistically significant for overweight or obesity and unhealthy diet, recognition of these factors was also suboptimal across age groups. This suggests that younger adults may be insufficiently reached by cancer prevention messages, especially those linking everyday health behaviors with long-term cancer risk.

The contrast with tobacco smoking is also informative. Smoking tobacco was among the most frequently recognized cancer risk factors in our study, yet daily smoking prevalence in Poland remains higher than the OECD average [32]. This suggests that awareness is necessary but not sufficient for behavior change, particularly where addictive substances are involved. At the same time, the high recognition of tobacco-related cancer risk may reflect the cumulative effects of long-standing public health communication and regulatory measures, including health warnings on packaging, taxation, advertising restrictions, smoke-free policies, and public campaigns [33]. This experience may offer useful lessons for other modifiable cancer risk factors, particularly alcohol use, physical inactivity, excess body weight, and unhealthy dietary patterns, where both population exposure and awareness gaps remain substantial.

Women and respondents with higher education demonstrated significantly greater awareness of most risk factors, replicating the pattern observed in the Polish genitourinary cancer risk factor awareness study [23], where better-educated and female respondents more frequently identified established risk determinants. Importantly, prior research from high-income countries has shown that knowledge of cancer risk factors is positively associated with engagement in risk reduction behaviors, such as smoking cessation, limiting alcohol intake, maintaining a healthy body weight, and participating in screening. Importantly, knowledge of cancer risk factors has been positively associated with engagement in risk-reduction behavior in high-income countries [34], underscoring the public health significance of these gaps.

### Limitations

Several limitations of the study should be acknowledged. The CAWI methodology may underrepresent older adults and individuals with lower educational attainment, potentially leading to an overestimation of cancer awareness in the general population. Non-probability quota sampling was applied, which, although widely used in population-based research in Poland, does not allow calculation of response rates in the traditional sense. Moreover, non-probability quota sampling may have introduced selection bias and may limit the generalizability of the findings. All variables, including cancer history and family history of cancer, were self-reported and may be subject to recall bias. The questionnaire assessed selected aspects of cancer knowledge and awareness rather than providing a comprehensive evaluation of all cancer-related knowledge domains. Because multiple statistical comparisons were performed, the possibility of Type I error cannot be excluded, and the findings from Table 4, Table 5 and Table 6 should be interpreted with appropriate caution. Furthermore, the analyses presented in Table 4, Table 5 and Table 6 were exploratory, and residual confounding cannot be excluded. The cross-sectional design precludes causal inference regarding the identified associations. Self-reported knowledge may not accurately reflect respondents’ actual knowledge and should therefore be interpreted with caution.

## 5. Conclusions

Public awareness of cancer warning signs, risk factors, and prevention methods among adults in Poland remains limited. Awareness was consistently lower among men, younger adults, and individuals with lower educational attainment. Personal and family history of cancer were associated with higher self-reported cancer knowledge. These findings support the need for targeted educational initiatives to improve awareness of cancer symptoms, risk factors, and evidence-based prevention strategies, particularly among demographic groups with lower levels of awareness.

## Figures and Tables

**Table 1:** Characteristics of the study population (n = 1087).

Variable	n	%
**Gender**		
female	551	50.7
male	536	49.3
**Age [years]**		
18–29	134	12.3
30–39	200	18.4
40–49	217	20.0
50–59	205	18.9
60+	331	30.5
**Educational level**		
higher	485	44.6
less than higher	602	55.4
**Marital status**		
single	313	28.8
married	570	52.4
informal relationship	157	14.4
other	47	4.3
**Place of residence**		
rural area	436	40.1
city below 20,000 residents	133	12.2
city from 20,000 to 99,999 residents	203	18.7
city from 100,000 to 499,999 residents	174	16.0
city ≥ 500,000 residents	141	13.0
**Number of household members**		
1 (living alone)	182	16.7
2	399	36.7
3 or more	506	46.6
**Having children**		
yes	717	66.0
no	370	34.0
**Occupational status**		
active	673	61.9
passive	414	38.1
**Have you ever been diagnosed with cancer (any cancer) by a physician?**		
yes	144	13.2
no	904	83.2
prefer not to answer	39	3.6
**Has anyone in your immediate family (e.g., parents, siblings, grandparents, or children) ever had cancer?**		
yes	542	49.9
no	385	35.4
I do not know	132	12.1
prefer not to answer	28	2.6

**Table 2:** Public knowledge of cancers (oncological diseases) among adults in Poland (n = 1087).

Variable	n	%
**How** **would you rate your knowledge of cancer (oncological diseases)?**		
very low	95	8.7
rather low	285	26.2
average	569	52.3
rather high	105	9.7
very high	33	3.0
**Which of the following do you think may be early warning signs of cancer?** *Select all that apply.*		
Unexplained weight loss (more than 5–10% of body weight)	648	59.6
Persistent fatigue and weakness despite adequate rest	523	48.1
Long-lasting, unusual, or worsening pain	545	50.1
Changes in skin appearance (e.g., a new mole, changes in an existing mole, non-healing skin lesions)	643	59.2
Enlarged lymph nodes	654	60.2
A lump, mass, thickening, or newly developed asymmetry	727	66.9
Changes in bowel or bladder habits, including blood in the urine	544	50.0
Persistent cough or hoarseness lasting longer than 3–6 weeks	591	54.4
Difficulty swallowing	395	36.3
Unusual bleeding or discharge	563	51.8
Wounds or ulcers that do not heal	450	41.4
Night sweats and prolonged fever	371	34.1
None of the above	22	2.0
Difficult to say	155	14.3
**In your opinion, which of the following factors may increase the risk of developing cancer?** *Select all that apply.*		
Tobacco use (cigarettes, pipe, cigars)	705	64.9
Alcohol consumption	527	48.5
Overweight or obesity	455	41.9
Low level of physical activity (sedentary lifestyle)	412	37.9
Unhealthy diet (e.g., low in fruits and vegetables, high in meat and ultra-processed foods)	496	45.6
Excessive exposure to ultraviolet (UV) radiation (sunlight, tanning beds)	643	59.2
Human papillomavirus (HPV) infection	524	48.2
Chronic hepatitis B or hepatitis C virus infection	446	41.0
Helicobacter pylori infection	321	29.5
Occupational or environmental exposure to carcinogens (e.g., asbestos, radon, air pollution, chemicals)	622	57.2
Hormonal factors (e.g., early menarche, late menopause, nulliparity, late age at first childbirth, polycystic ovary syndrome, testosterone supplementation)	391	36.0
Genetic or familial predisposition (e.g., mutations in certain genes)	672	61.8
Chronic inflammation	489	45.0
None of the above	23	2.1
Difficult to say	144	13.2
**In your opinion, how can cancer be prevented or its risk reduced?** *Select all methods that you believe may help prevent cancer.*		
Quitting smoking and avoiding exposure to secondhand smoke	625	57.5
Limiting alcohol consumption or abstaining from alcohol	513	47.2
Maintaining a healthy body weight (avoiding overweight and obesity)	433	39.8
Regular physical activity (e.g., at least 150 min of moderate-intensity activity per week)	475	43.7
A healthy diet rich in vegetables, fruits, whole grains, and legumes	515	47.4
Limiting consumption of red and processed meat and ultra-processed foods	396	36.4
Reducing intake of sugar, sweets, and sugar-sweetened beverages	440	40.5
Avoiding excessive UV exposure (sunlight, tanning beds) and using sun protection	567	52.2
Regular participation in preventive health examinations and cancer screening programs (e.g., cervical cytology, mammography, colonoscopy)	625	57.5
Vaccination (e.g., against HPV and hepatitis B), when recommended	455	41.9
Seeking medical attention when experiencing concerning symptoms (e.g., persistent cough, lumps, abnormal bleeding)	617	56.8
Avoiding exposure to carcinogenic substances at work and using appropriate personal protective equipment	563	51.8
Reducing air pollution exposure whenever possible (e.g., avoiding smoking indoors)	481	44.3
Breastfeeding, if possible	167	15.4
Appropriate use of hormone replacement therapy and hormonal contraception in accordance with medical advice	274	25.2
Difficult to say	152	14.0
*Incorrect answers*		
There are no actions that can reduce the risk of cancer	35	3.2
Regular use of dietary supplements (e.g., vitamins, herbal products) protects against cancer	226	20.8
Drinking “detox” beverages, special juices, or teas prevents cancer	176	16.2
None of the above	22	2.0

**Table 3:** Factors associated with self-reported knowledge of cancers among adults in Poland (n = 1087).

How Would You Rate Your Knowledge Level About Cancer (Oncological Diseases)?—Rather High or Very High						
	%	*p*	Bivariable Logistic Regression		Multivariable Logistic Regression	
Variable	n	%	OR (95%CI)	*p*	aOR (95%CI)	*p*
**Gender**						
female (n = 551)	13.8	0.3	1.22 (0.85–1.75)	0.3		
male (n = 536)	11.6		Reference			
**Age [years]**						
18–29 (n = 134)	9.0	0.2	Reference			
30–39 (n = 200)	16.5		2.01 (0.99–4.05)	0.05		
40–49 (n = 217)	12.4		1.45 (0.71–2.96)	0.3		
50–59 (n = 205)	15.1		1.81 (0.90–3.67)	0.1		
60+ (n = 331)	10.6		1.20 (0.60–2.39)	0.6		
**Educational level**						
higher (n = 485)	16.1	**0.003**	1.73 (1.21–2.48)	**0.003**	1.67 (1.13–2.46)	**0.009**
less than higher (n = 602)	10.0		Reference		Reference	
**Marital status**						
single (n = 313)	11.2	0.08	Reference		Reference	
married (n = 570)	11.9		1.08 (0.70–1.66)	0.7	0.93 (0.59–1.46)	0.7
informal relationship (n = 157)	15.3		1.43 (0.82–2.51)	0.2	1.41 (0.78–2.53)	0.3
other (divorced or widowed) (n = 47)	23.4		2.43 (1.13–5.20)	**0.02**	2.97 (1.32–6.69)	**0.008**
**Place of residence**						
rural area (n = 436)	9.2	**0.03**	Reference		Reference	
city below 20,000 residents (n = 133)	14.3		1.65 (0.92–2.96)	0.09	1.54 (0.83–2.84)	0.2
city from 20,000 to 99,999 residents (n = 203)	16.7		1.99 (1.22–3.26)	**0.006**	1.58 (0.94–2.65)	0.1
city from 100,000 to 499,999 residents (n = 174)	16.7		1.98 (1.18–3.31)	**0.009**	1.54 (0.89–2.64)	0.1
city ≥ 500,000 residents (n = 141)	11.3		1.27 (0.69–2.34)	0.4	1.09 (0.58–2.06)	0.8
**Number of household members**						
1 (living alone) (n = 182)	13.2	0.9	Reference			
2 (n = 399)	12.5		0.94 (0.56–1.59)	0.8		
3 or more (n = 506)	12.6		0.95 (0.58–1.58)	0.9		
**Having children**						
yes (n = 717)	13.2	0.4	1.16 (0.79–1.71)	0.4		
no (n = 370)	11.6		Reference			
**Occupational status**						
active (n = 673)	14.3	**0.04**	1.47 (1.01–2.17)	**0.04**	1.56 (1.02–2.37)	**0.04**
passive (n = 414)	10.1		Reference		Reference	
**Have you ever been diagnosed with cancer (any type of cancer) by a physician?**						
yes (n = 144)	31.3	**<0.001**	4.15 (2.75–6.27)	**<0.001**	4.27 (2.75–6.64)	**<0.001**
no (n = 904)	9.8		Reference *		Reference *	
prefer not to answer (n = 39)	10.3					
**Has anyone in your immediate family (e.g., parents, siblings, grandparents, or children) ever had cancer?**						
yes (n = 542)	16.8	**<0.001**	2.14 (1.47–3.11)	**<0.001**	1.85 (1.25–2.73)	**0.002**
no (n = 385)	8.8		Reference **		Reference **	
I do not know (n = 132)	8.3					
prefer not to answer (n = 28)	7.1					

* Responses no or prefer not to answer were combined into one category; ** responses no, I do not know and prefer not to answer were combined into one category.

**Table 4:** Socio-demographic differences in public awareness of potential early warning signs of cancer among adults in Poland (n = 1087).

Which of the following do you think may be early warning signs of cancer?												
	Unexplained weight loss (more than 5–10% of body weight)		Persistent fatigue and weakness despite adequate rest		Long-lasting, unusual, or worsening pain		Changes in skin appearance		Enlarged lymph nodes		A lump, mass, thickening, or newly developed asymmetry	
	**%**	***p***	**%**	***p***	**%**	***p***	**%**	***p***	**%**	***p***	**%**	***p***
**Gender**												
female (n = 551)	69.5	**<0.001**	56.3	**<0.001**	58.4	**<0.001**	67.2	**<0.001**	70.4	**<0.001**	76.0	**<0.001**
male (n = 536)	49.4		39.7		41.6		50.9		49.6		57.5	
**Age [years]**												
18–29 (n = 134)	42.5	**<0.001**	41.8	**0.02**	44.8	0.3	44.0	**<0.001**	39.6	**<0.001**	51.5	**<0.001**
30–39 (n = 200)	50.5		43.5		48.0		49.5		51.0		58.0	
40–49 (n = 217)	59.4		44.7		49.3		57.6		58.5		65.9	
50–59 (n = 205)	69.3		56.6		56.6		64.9		64.9		75.6	
60+ (n = 331)	66.2		50.5		50.2		68.6		72.2		73.7	
**Educational level**												
higher (n = 485)	67.2	**<0.001**	56.5	**<0.001**	58.1	**<0.001**	68.9	**<0.001**	69.1	**<0.001**	75.5	**<0.001**
less than higher (n = 602)	53.5		41.4		43.7		51.3		53.0		60.0	
**Marital status**												
single (n = 313)	52.7	**0.01**	44.1	0.4	49.2	0.8	54.3	0.07	54.3	0.05	63.9	0.3
married (n = 570)	63.5		50.2		49.6		62.6		63.0		69.3	
informal relationship (n = 157)	56.7		49.0		54.1		55.4		59.2		63.7	
other (n = 47)	68.1		46.8		48.9		61.7		68.1		68.1	
**Place of residence**												
rural area (n = 436)	52.1	**<0.001**	42.9	**0.03**	45.6	0.1	53.0	**0.02**	53.9	**0.01**	59.9	**0.002**
city below 20,000 residents (n = 133)	57.9		48.9		53.4		63.2		64.7		71.4	
city from 20,000 to 99,999 residents (n = 203)	69.0		48.8		54.2		63.5		67.0		74.4	
city from 100,000 to 499,999 residents (n = 174)	65.5		56.3		49.4		61.5		62.1		69.5	
city ≥ 500,000 residents (n = 141)	63.8		52.5		56.0		65.2		63.1		70.2	
**Number of household members**												
1 (living alone) (n = 182)	54.9	0.09	42.9	0.2	48.9	0.8	54.9	**0.006**	56.6	**<0.001**	61.5	0.06
2 (n = 399)	63.7		50.9		51.4		65.4		67.4		70.9	
3 or more (n = 506)	58.1		47.8		49.6		55.7		55.7		65.6	
**Having children**												
yes (n = 717)	64.7	**<0.001**	50.2	0.05	51.5	0.2	63.9	**<0.001**	65.4	**<0.001**	71.0	**<0.001**
no (n = 370)	49.7		44.1		47.6		50.0		50.0		58.9	
**Occupational status**												
active (n = 673)	59.6	0.9	48.3	0.9	51.1	0.4	58.2	0.4	58.4	0.1	66.0	0.4
passive (n = 414)	59.7		47.8		48.6		60.6		41.6		68.4	
**Have you ever been diagnosed with cancer (any type of cancer) by a physician?**												
yes (n = 144)	60.4	**<0.001**	50.0	**0.006**	44.4	0.002	58.3	**<0.001**	57.6	**<0.001**	61.8	**<0.001**
no (n = 904)	61.3		48.9		52.1		61.1		62.5		69.6	
prefer not to answer (n = 39)	17.9		23.1		25.6		17.9		15.4		23.1	
**Has anyone in your immediate family (e.g., parents, siblings, grandparents, or children) ever had cancer?**												
yes (n = 542)	67.9	**<0.001**	42.1	**<0.001**	57.4	**<0.001**	70.1	**<0.001**	69.7	**<0.001**	76.8	**<0.001**
no (n = 385)	56.1		56.6		46.0		53.8		56.1		61.8	
I do not know (n = 132)	46.2		38.6		40.2		40.9		43.9		53.8	
prefer not to answer (n = 28)	10.7		10.7		14.3		7.1		7.1		7.1	
**Which** **of the following do you think may be early warning signs of cancer?**												
	Changes in bowel or bladder habits, including blood in the urine		Persistent cough or hoarseness lasting longer than 3–6 weeks		Difficulty swallowing		Unusual bleeding or discharge		Wounds or ulcers that do not heal		Night sweats and prolonged fever	
	**%**	***p***	**%**	***p***	**%**	***p***	**%**	***p***	**%**	***p***	**%**	***p***
**Gender**												
female (n = 551)	56.8	**<0.001**	63.3	**<0.001**	42.8	**<0.001**	59.7	**<0.001**	48.1	**<0.001**	40.7	**<0.001**
male (n = 536)	43.1		36.7		29.7		43.7		34.5		27.4	
**Age [years]**												
18–29 (n = 134)	29.1	**<0.001**	33.6	**<0.001**	26.9	0.2	40.3	**0.003**	35.1	**0.04**	29.1	0.8
30–39 (n = 200)	42.5		49.0		36.5		45.0		37.5		35.0	
40–49 (n = 217)	49.3		54.4		36.9		53.9		37.8		34.6	
50–59 (n = 205)	58.5		64.4		40.0		57.6		43.9		35.6	
60+ (n = 331)	58.3		59.8		37.5		55.6		47.1		34.4	
**Educational level**												
higher (n = 485)	56.9	**<0.001**	62.7	**<0.001**	43.1	**<0.001**	59.0	**<0.001**	48.7	**<0.001**	40.8	**<0.001**
less than higher (n = 602)	44.5		47.7		30.9		46.0		35.5		28.7	
**Marital status**												
single (n = 313)	43.1	**0.02**	48.6	0.04	31.3	0.1	42.5	**<0.001**	36.7	0.2	29.1	0.07
married (n = 570)	54.0		58.1		39.5		56.1		44.2		36.3	
informal relationship (n = 157)	49.0		51.6		34.4		51.6		39.5		33.1	
other (n = 47)	51.1		57.4		38.3		61.7		44.7		44.7	
**Place of residence**												
rural area (n = 436)	43.1	**0.006**	47.7	**0.005**	32.6	0.1	49.1	0.2	39.0	0.06	31.0	0.4
city below 20,000 residents (n = 133)	52.6		54.1		37.6		47.4		37.6		33.8	
city from 20,000 to 99,999 residents (n = 203)	55.7		61.6		35.5		58.1		42.4		38.4	
city from 100,000 to 499,999 residents (n = 174)	53.4		60.3		40.2		51.7		40.2		34.5	
city ≥ 500,000 residents (n = 141)	56.7		57.4		43.3		55.3		52.5		37.6	
**Number of household members**												
1 (living alone) (n = 182)	45.1	**0.003**	51.6	0.07	25.3	**0.003**	45.6	0.1	36.8	0.4	25.3	**0.02**
2 (n = 399)	56.9		58.9		38.6		54.4		43.1		37.1	
3 or more (n = 506)	46.4		51.8		38.5		52.0		41.7		35.0	
**Having children**												
yes (n = 717)	55.1	**<0.001**	58.7	**<0.001**	38.9	**0.01**	56.2	**<0.001**	44.2	**0.009**	35.1	0.3
no (n = 370)	40.3		45.9		31.4		43.2		35.9		32.2	
**Occupational status**												
active (n = 673)	49.3	0.5	55.0	0.6	37.3	0.4	53.8	0.09	40.9	0.6	35.7	0.2
passive (n = 414)	51.2		53.4		34.8		46.2		42.3		31.6	
**Have you ever been diagnosed with cancer (any type of cancer) by a physician?**												
yes (n = 144)	54.2	**<0.001**	56.3	**<0.001**	36.8	**0.008**	47.9	**<0.001**	41.7	**0.001**	42.4	**0.01**
no (n = 904)	50.7		55.5		37.3		53.9		42.6		33.5	
prefer not to answer (n = 39)	20.5		20.5		12.8		17.9		12.8		17.9	
**Has anyone in your immediate family (e.g., parents, siblings, grandparents, or children) ever had cancer?**												
yes (n = 542)	58.1	**<0.001**	62.4	**<0.001**	43.4	**<0.001**	60.7	**<0.001**	46.7	**<0.001**	40.4	**<0.001**
no (n = 385)	45.7		50.4		31.9		47.3		40.0		29.6	
I do not know (n = 132)	37.9		40.9		25.8		36.4		30.3		26.5	
prefer not to answer (n = 28)	10.7		17.9		10.7		14.3		10.7		10.7	

**Table 5:** Socio-demographic differences in public awareness of cancer risk factors among adults in Poland (n = 1087).

In your opinion, which of the following factors may increase the risk of developing cancer?														
			Tobacco use (cigarettes, pipe, cigars)		Alcohol consumption		Overweight or obesity		Low level of physical activity (sedentary lifestyle)		Unhealthy diet		Excessive exposure to ultraviolet (UV) radiation (sunlight, tanning beds)	
			**%**	***p***	**%**	***p***	**%**	***p***	**%**	***p***	**%**	***p***	**%**	***p***
**Gender**														
female (n = 551)			69.7	**<0.001**	50.5	0.2	42.3	0.8	38.8	0.5	50.1	**0.003**	65.7	**<0.001**
male (n = 536)			59.9		46.5		41.4		36.9		41.0		52.4	
**Age [years]**														
18–29 (n = 134)			51.5	**<0.001**	35.8	**0.02**	35.1	0.3	29.9	**0.02**	41.0	0.6	44.0	**<0.001**
30–39 (n = 200)			55.5		47.0		39.4		31.5		42.5		47.0	
40–49 (n = 217)			61.8		49.3		46.1		43.8		47.9		58.5	
50–59 (n = 205)			69.8		50.7		41.5		41.5		47.8		62.9	
60+ (n = 331)			74.9		52.6		43.5		39.0		46.5		70.7	
**Educational level**														
higher (n = 485)			72.8	**<0.001**	56.1	**<0.001**	48.7	**<0.001**	47.2	**<0.001**	54.6	**<0.001**	67.2	**<0.001**
less than higher (n = 602)			58.5		42.4		36.4		30.4		38.4		52.7	
**Marital status**														
single (n = 313)			61.3	0.2	45.7	0.6	36.7	0.1	33.9	0.3	42.2	0.3	55.0	0.3
married (n = 570)			67.4		49.1		44.0		40.2		47.4		61.8	
informal relationship (n = 157)			65.0		50.3		45.2		38.2		48.4		58.6	
other (n = 47)			57.4		53.2		38.3		36.2		38.3		57.4	
**Place of residence**														
rural area (n = 436)			58.3	**0.002**	40.4	**<0.001**	36.7	**0.03**	32.6	**0.04**	37.8	**<0.001**	53.0	**0.02**
city below 20,000 residents (n = 133)			63.9		53.4		40.6		38.3		43.6		60.9	
city from 20,000 to 99,999 residents (n = 203)			72.9		57.6		47.3		43.8		52.2		64.5	
city from 100,000 to 499,999 residents (n = 174)			69.0		48.3		43.1		39.7		52.9		62.1	
city ≥ 500,000 residents (n = 141)			69.5		56.0		49.6		43.3		53.2		65.2	
**Number of household members**														
1 (living alone) (n = 182)			60.4	**0.009**	45.6	0.1	31.3	**0.006**	31.9	0.1	40.7	0.2	57.7	**0.005**
2 (n = 399)			70.7		52.4		44.6		41.1		48.4		65.4	
3 or more (n = 506)			61.9		46.4		43.5		37.5		45.3		54.7	
**Having children**														
yes (n = 717)			69.0	**<0.001**	50.9	**0.03**	42.5	0.5	39.2	0.2	47.3	0.1	63.0	**<0.001**
no (n = 370)			56.8		43.8		40.5		35.4		42.4		51.6	
**Occupational status**														
active (n = 673)			63.4	0.2	48.6	0.9	42.6	0.5	39.1	0.3	46.5	0.5	56.8	**0.04**
passive (n = 414)			67.1		48.3		40.6		36.0		44.2		63.0	
**Have you ever been diagnosed with cancer (any type of cancer) by a physician?**														
yes (n = 144)			60.4	**<0.001**	46.5	**0.01**	42.6	**0.02**	40.3	**0.01**	48.6	**0.002**	54.2	**<0.001**
no (n = 904)			67.1		49.8		43.1		38.5		46.3		61.7	
prefer not to answer (n = 39)			28.2		25.6		20.5		15.4		17.9		17.9	
**Has anyone in your immediate family (e.g., parents, siblings, grandparents, or children) ever had cancer?**														
yes (n = 542)			74.2	**<0.001**	54.8	**<0.001**	40.8	**0.02**	43.4	**<0.001**	42.1	**<0.001**	67.2	**<0.001**
no (n = 385)			59.0		45.2		44.8		34.0		53.3		55.8	
I do not know (n = 132)			53.8		39.4		37.9		33.3		31.8		46.2	
prefer not to answer (n = 28)			17.9		14.3		17.9		7.1		10.7		10.7	
**In your opinion, which of the following factors may increase the risk of developing cancer?**														
	Human papillomavirus (HPV) infection		Chronic hepatitis B or hepatitis C virus infection		Helicobacter pylori infection		Occupational or environmental exposure to carcinogens		Hormonal factors		Genetic or familial predisposition		Chronic inflammation	
	**%**	***p***	**%**	***p***	**%**	***p***	**%**	***p***	**%**	***p***	**%**	***p***	**%**	***p***
**Gender**														
female (n = 551)	57.0	**<0.001**	47.9	**<0.001**	33.8	**0.002**	65.5	**<0.001**	42.3	**<0.001**	70.4	**<0.001**	51.9	**<0.001**
male (n = 536)	39.2		34.0		25.2		48.7		29.5		53.0		37.9	
**Age [years]**														
18–29 (n = 134)	36.6	**<0.001**	29.9	**0.04**	22.4	0.2	40.3	**<0.001**	36.6	**0.02**	47.0	**<0.001**	26.9	**<0.001**
30–39 (n = 200)	41.5		39.0		27.0		51.0		36.0		51.5		45.5	
40–49 (n = 217)	46.1		41.0		31.8		56.2		41.9		59.0		44.2	
50–59 (n = 205)	54.6		44.9		32.2		62.9		40.5		70.2		48.3	
60+ (n = 331)	54.4		44.4		30.8		65.0		29.0		70.7		50.5	
**Educational level**														
higher (n = 485)	57.1	**<0.001**	48.7	**<0.001**	38.8	**<0.001**	66.6	**<0.001**	44.9	**<0.001**	71.3	**<0.001**	53.8	**<0.001**
less than higher (n = 602)	41.0		34.9		22.1		49.7		28.7		54.2		37.9	
**Marital status**														
single (n = 313)	40.3	**0.007**	39.9	0.4	25.2	0.2	56.5	0.9	33.5	0.6	55.6	**0.04**	40.3	0.1
married (n = 570)	51.4		41.2		30.7		58.2		37.9		65.3		47.7	
informal relationship (n = 157)	53.5		45.2		31.8		54.8		35.0		61.8		42.7	
other (n = 47)	44.7		31.9		36.2		57.4		31.9		61.7		51.1	
**Place of residence**														
rural area (n = 436)	42.4	**0.02**	31.7	**<0.001**	23.9	**0.009**	47.0	**<0.001**	31.7	0.1	55.7	**0.02**	40.6	0.2
city below 20,000 residents (n = 133)	52.6		47.4		33.1		60.9		34.6		65.4		45.9	
city from 20,000 to 99,999 residents (n = 203)	49.3		49.8		31.5		66.5		38.9		65.0		50.7	
city from 100,000 to 499,999 residents (n = 174)	51.7		45.4		31.6		63.2		39.7		65.5		46.0	
city ≥ 500,000 residents (n = 141)	56.0		46.1		38.3		64.5		41.8		68.1		48.2	
**Number of household members**														
1 (living alone) (n = 182)	42.9	**0.01**	41.2	0.7	26.4	0.05	56.6	0.09	27.5	**0.002**	61.0	0.2	41.8	0.3
2 (n = 399)	54.1		42.6		31.1		61.4		33.3		65.4		48.1	
3 or more (n = 506)	45.5		39.7		29.4		54.2		41.1		59.3		43.7	
**Having children**														
yes (n = 717)	52.0	**<0.001**	42.8	0.1	31.7	**0.03**	61.6	**<0.001**	36.7	0.5	66.1	**<0.001**	48.0	**0.006**
no (n = 370)	40.8		37.6		25.4		48.6		34.6		53.5		39.2	
**Occupational status**														
active (n = 673)	48.3	0.9	41.9	0.5	32.4	**0.008**	54.5	**0.02**	40.4	**<0.001**	60.6	0.3	45.2	0.9
passive (n = 414)	48.1		39.6		24.9		61.6		28.7		63.8		44.7	
**Have you ever been diagnosed with cancer (any type of cancer) by a physician?**														
yes (n = 144)	45.1	**<0.001**	38.2	**0.007**	25.7	**0.01**	61.8	**<0.001**	34.7	**0.02**	54.9	**<0.001**	43.1	**<0.001**
no (n = 904)	50.0		42.5		31.0		58.3		37.1		64.7		46.6	
prefer not to answer (n = 39)	17.9		17.9		10.3		15.4		15.4		20.5		15.4	
**Has anyone in your immediate family (e.g., parents, siblings, grandparents, or children) ever had cancer?**														
yes (n = 542)	51.8	**<0.001**	40.0	**0.001**	32.5	**0.01**	64.0	**<0.001**	42.3	**<0.001**	56.9	**<0.001**	49.6	**<0.001**
no (n = 385)	49.4		45.4		29.6		54.0		31.2		70.7		43.4	
I do not know (n = 132)	38.6		31.1		21.2		49.2		28.0		51.5		37.9	
prefer not to answer (n = 28)	7.1		17.9		10.7		7.1		17.9		7.1		10.7	

**Table 6:** Socio-demographic differences in public awareness of cancer prevention methods among adults in Poland (n = 1087).

In your opinion, how can cancer be prevented or its risk reduced?																		
	Quitting smoking and avoiding exposure to secondhand smoke		Limiting alcohol consumption or abstaining from alcohol		Maintaining a healthy body weight		Regular physical activity		A healthy diet rich in vegetables, fruits, whole grains, and legumes		Limiting consumption of red and processed meat and ultra-processed foods		Reducing intake of sugar, sweets, and sugar-sweetened beverages		Avoiding excessive UV exposure (sunlight, tanning beds) and using sun protection		Regular participation in preventive health examinations and cancer screening programs	
	**%**	***p***	**%**	***p***	**%**	***p***	**%**	***p***	**%**	***p***	**%**	***p***	**%**	***p***	**%**	***p***	**%**	***p***
**Gender**																		
female (n = 551)	63.4	**<0.001**	52.5	**<0.001**	43.2	0.02	46.1	0.1	52.8	**<0.001**	41.4	**<0.001**	43.7	**0.03**	60.3	**<0.001**	65.3	**<0.001**
male (n = 536)	52.4		41.8		36.4		41.2		41.8		31.3		37.1		43.8		49.4	
**Age [years]**																		
18–29 (n = 134)	44.8	**<0.001**	38.1		32.1	0.03	41.0	0.5	37.3	**<0.001**	29.1	0.1	29.1	**0.03**	38.8	**<0.001**	44.0	**<0.001**
30–39 (n = 200)	46.5		43.5		34.0		39.0		40.0		32.5		39.0		44.0		44.0	
40–49 (n = 217)	55.4		46.1		40.1		45.6		45.2		36.9		39.2		47.9		53.9	
50–59 (n = 205)	61.0		50.7		41.0		46.8		50.7		38.5		44.9		55.6		62.9	
60+ (n = 331)	68.6		51.7		45.6		44.4		55.3		40.2		44.1		63.1		70.1	
**Educational level**																		
higher (n = 485)	66.8	**<0.001**	57.3	**<0.001**	49.1	**<0.001**	52.8	**<0.001**	57.7	**<0.001**	46.4	**<0.001**	50.9	**<0.001**	59.8	**<0.001**	65.8	**<0.001**
less than higher (n = 602)	50.0		39.0		32.4		36.4		39.0		28.4		32.1		46.0		50.8	
**Marital status**																		
single (n = 313)	54.6	0.6	42.5	0.2	35.1	0.2	38.7	0.2	42.8	0.2	34.2	0.7	34.2	0.06	49.2	0.3	52.1	**0.04**
married (n = 570)	58.8		48.6		41.1		45.4		48.4		37.0		42.6		54.4		61.4	
informal relationship (n = 157)	59.2		49.7		43.3		47.1		51.0		36.9		43.3		52.9		53.5	
other (n = 47)	55.3		53.2		44.7		44.7		53.2		42.6		46.8		42.6		59.6	
**Place of residence**																		
rural area (n = 436)	49.3	**<0.001**	40.6	**0.002**	35.1	0.06	39.0	**<0.001**	40.8	**0.004**	28.7	**<0.001**	35.1	**0.01**	45.0	**0.003**	51.6	**0.007**
city below 20,000 residents (n = 133)	60.9		48.9		37.6		41.4		48.1		33.8		43.6		54.9		59.4	
city from 20,000 to 99,999 residents (n = 203)	66.0		56.7		45.8		58.1		56.7		46.8		49.3		59.6		67.0	
city from 100,000 to 499,999 residents (n = 174)	63.2		47.1		44.3		43.7		50.0		38.5		38.5		55.2		59.2	
city ≥ 500,000 residents (n = 141)	60.3		52.5		42.6		39.7		50.4		45.4		44.0		57.4		58.2	
**Number of household members**																		
1 (living alone) (n = 182)	56.0	**<0.001**	43.4	0.2	38.5	0.1	37.9	0.2	45.1	**0.03**	36.3	0.2	34.1	**0.04**	51.1	0.3	54.9	0.06
2 (n = 399)	65.2		50.9		43.9		45.1		52.6		39.6		44.9		55.4		62.2	
3 or more (n = 506)	52.0		45.7		37.2		44.7		44.1		34.0		39.3		50.0		54.7	
**Having children**																		
yes (n = 717)	60.7	**0.003**	50.6	**0.002**	41.7	0.08	45.5	0.1	50.3	**0.006**	38.5	**0.04**	44.1	**<0.001**	56.3	**<0.001**	63.0	**<0.001**
no (n = 370)	51.4		40.5		36.2		40.3		41.6		32.4		33.5		44.1		46.8	
**Occupational status**																		
active (n = 673)	54.8	**0.02**	45.9	0.3	38.5	0.2	44.7	0.4	46.2	0.3	36.6	0.9	41.2	0.6	49.8	**0.04**	54.4	**0.008**
passive (n = 414)	61.8		49.3		42.0		42.0		49.3		36.2		39.4		56.0		62.6	
**Have you ever been diagnosed with cancer (any type of cancer) by a physician?**																		
yes (n = 144)	54.9	**<0.001**	50.0	**<0.001**	38.9	**0.002**	43.8	**0.03**	48.6	**0.003**	36.1	0.1	38.9	**0.01**	50.7	**<0.001**	56.3	**<0.001**
no (n = 904)	59.5		48.0		41.2		44.6		48.3		37.2		41.7		53.8		59.0	
prefer not to answer (n = 39)	20.5		17.9		12.8		23.1		20.5		20.5		17.9		20.5		28.2	
**Has anyone in your immediate family (e.g., parents, siblings, grandparents, or children) ever had cancer?**																		
yes (n = 542)	66.1	**<0.001**	56.3	**<0.001**	45.8	**<0.001**	51.5	**<0.001**	54.4	**<0.001**	42.6	**<0.001**	46.9	**<0.001**	60.1	**<0.001**	66.1	**<0.001**
no (n = 385)	53.2		42.1		36.6		38.4		42.9		34.8		37.9		48.6		52.2	
I do not know (n = 132)	44.7		33.3		32.6		34.1		39.4		22.7		28.0		40.2		47.7	
prefer not to answer (n = 28)	10.7		7.1		3.6		10.7		10.7		3.6		10.7		3.6		10.7	
**In** **your opinion, how can cancer be prevented or its risk reduced?**																		
	Vaccination (e.g., against HPV and hepatitis B), when recommended		Seeking medical attention when experiencing concerning symptoms		Avoiding exposure to carcinogenic substances at work and using appropriate personal protective equipment		Reducing air pollution exposure whenever possible (e.g., avoiding smoking indoors)		Breastfeeding, if possible		Appropriate use of hormone replacement therapy and hormonal contraception in accordance with medical advice		There are no actions that can reduce the risk of cancer		Regular use of dietary supplements (e.g., vitamins, herbal products) protects against cancer		Drinking “detox” beverages, special juices, or teas prevents cancer	
	**%**	***p***	**%**	***p***	**%**	***p***	**%**	***p***	**%**	***p***	**%**	***p***	**%**	***p***	**%**	***p***	**%**	***p***
**Gender**																		
female (n = 551)	46.5	**0.002**	64.6	**<0.001**	58.3	**<0.001**	49.4	**<0.001**	18.0	**0.02**	31.6	**<0.001**	3.1	0.8	22.3	0.2	17.1	0.4
male (n = 536)	37.1		48.7		45.1		39.0		12.7		18.7		3.4		19.2		15.3	
**Age [years]**																		
18–29 (n = 134)	33.6	**0.003**	43.3	**<0.001**	39.6	**<0.001**	35.1	**0.002**	6.7	**0.03**	22.4	0.9	2.2	0.9	20.9	0.3	11.9	0.6
30–39 (n = 200)	34.5		47.0		42.5		38.0		14.0		25.0		3.0		24.0		15.5	
40–49 (n = 217)	40.6		53.5		49.8		41.0		17.1		24.9		3.2		18.4		15.7	
50–59 (n = 205)	44.4		62.0		56.1		48.3		18.5		26.8		3.9		24.4		18.5	
60+ (n = 331)	48.9		67.1		61.0		51.4		16.6		25.7		3.3		18.1		17.2	
**Educational level**																		
higher (n = 485)	50.1	**<0.001**	63.5	**<0.001**	60.6	**<0.001**	52.8	**<0.001**	19.4	**<0.001**	33.6	**<0.001**	1.9	**0.02**	23.5	**0.04**	19.8	**0.004**
less than higher (n = 602)	35.2		51.3		44.7		37.4		12.1		18.4		4.3		18.6		13.3	
**Marital status**																		
single (n = 313)	36.1	0.1	54.0	0.1	52.1	0.8	41.2	0.6	9.9	**0.008**	22.0	0.4	2.2	0.6	19.2	0.3	15.3	0.3
married (n = 570)	44.4		59.5		52.8		45.4		18.1		26.3		3.3		20.0		16.5	
informal relationship (n = 157)	44.6		50.3		48.4		45.2		14.6		25.5		4.5		26.8		14.0	
other (n = 47)	40.4		63.8		48.9		46.8		21.3		31.9		4.3		21.3		25.5	
**Place of residence**																		
rural area (n = 436)	35.3	**0.008**	52.3	0.06	45.6	**0.003**	38.5	**0.01**	13.1	0.4	21.3	0.06	3.2	**0.02**	18.6	0.5	12.6	**0.04**
city below 20,000 residents (n = 133)	48.1		56.4		48.9		41.4		15.0		27.8		0.8		19.5		13.5	
city from 20,000 to 99,999 residents (n = 203)	47.3		64.5		60.6		48.3		16.3		29.6		1.5		24.6		19.2	
city from 100,000 to 499,999 residents (n = 174)	43.1		59.2		54.0		51.1		17.2		23.0		4.0		21.3		20.7	
city ≥ 500,000 residents (n = 141)	46.8		56.7		58.2		50.4		19.1		31.2		7.1		22.7		19.9	
**Number of household members**																		
1 (living alone) (n = 182)	40.1	0.3	57.1	0.2	52.7	0.05	42.3	0.3	9.9	0.06	20.3	0.3	3.3	0.7	15.4	**0.03**	14.3	0.7
2 (n = 399)	45.1		60.2		56.1		47.1		15.3		26.3		3.8		19.0		16.8	
3 or more (n = 506)	39.9		54.0		48.0		42.7		17.4		26.1		2.8		24.1		16.4	
**Having children**																		
yes (n = 717)	44.8	**0.007**	60.3	**0.001**	55.6	**<0.001**	47.1	**0.008**	18.3	**<0.001**	26.8	0.1	3.8	0.2	21.1	0.8	17.0	0.3
no (n = 370)	36.2		50.0		44.3		38.6		9.7		22.2		2.2		20.3		14.6	
**Occupational status**																		
active (n = 673)	40.0	0.1	54.5	0.06	50.1	0.1	43.1	0.3	15.8	0.7	27.3	**0.04**	3.1	0.8	23.3	**0.01**	16.6	0.6
passive (n = 414)	44.9		60.4		54.6		46.1		14.7		21.7		3.4		16.7		15.5	
**Have you ever been diagnosed with cancer (any type of cancer) by a physician?**																		
yes (n = 144)	43.1	**0.003**	56.9	**<0.001**	50.0	**<0.001**	45.1	**0.001**	18.8	0.1	23.6	0.2	1.4	0.3	22.2	0.2	16.7	0.2
no (n = 904)	42.8		58.4		53.3		45.4		15.3		26.0		3.4		21.0		16.6	
prefer not to answer (n = 39)	15.4		17.9		23.1		15.4		5.1		12.8		5.1		10.3		5.1	
**Has anyone in your immediate family (e.g., parents, siblings, grandparents, or children) ever had cancer?**																		
yes (n = 542)	49.1	**<0.001**	64.4	**<0.001**	61.3	**<0.001**	39.2	**<0.001**	16.2		28.0	**0.007**	1.5	**0.01**	22.9	0.09	17.7	0.06
no (n = 385)	37.1		53.5		46.5		52.6		16.6		24.9		4.7		20.8		16.9	
I do not know (n = 132)	34.1		44.7		37.9		33.3		10.6		18.9		5.3		14.4		10.6	
prefer not to answer (n = 28)	3.6		10.7		7.1		3.6		3.6		3.6		7.1		10.7		3.6	

## Data Availability

The data presented in this study are available on request from the corresponding author. The data are not publicly available due to privacy and institutional restrictions.
